# CB1 cannabinoid receptor-mediated plasticity of GABAergic synapses in the mouse insular cortex

**DOI:** 10.1038/s41598-020-64236-5

**Published:** 2020-04-28

**Authors:** Hiroki Toyoda

**Affiliations:** Department of Oral Physiology, Osaka University Graduate School of Dentistry, Suita Osaka, 565-0871 Japan

**Keywords:** Neuroscience, Physiology

## Abstract

The insular cortex plays pivotal roles in taste learning. As cellular mechanisms of taste learning, long-term potentiation (LTP) at glutamatergic synapses is well studied. However, little is known about long-term changes of synaptic efficacy at GABAergic synapses in the insular cortex. Here, we examined the synaptic mechanisms of long-term plasticity at GABAergic synapses in layer V pyramidal neurons of the mouse insular cortex. In response to a prolonged high-frequency stimulation (HFS), GABAergic synapses displayed endocannabinod (eCB)-mediated long-term depression (LTD_GABA_). When cannabinoid 1 receptors (CB1Rs) were blocked by a CB1R antagonist, the same stimuli caused LTP at GABAergic synapses (LTP_GABA_) which was mediated by production of nitric oxide (NO) via activation of NMDA receptors. Intriguingly, NO signaling was necessary for the induction of LTD_GABA_. In the presence of leptin which blocks CB1 signaling, the prolonged HFS caused LTP_GABA_ which was mediated by NO signaling. These results indicate that long-term plasticity at GABAergic synapses in the insular cortex can be modulated by combined effects of eCB and NO signaling. These forms of GABAergic synaptic plasticity in the insular cortex may be crucial synaptic mechanisms in taste learning.

## Introduction

The insular cortex plays critical roles in sensory and cognitive functions, such as pain perception, taste memory and interoceptive awareness^1–3^. The insular cortex can be generally divided into the gustatory and visceral insular cortex^4^. The gustatory insular cortex is associated with taste learning such as conditioned taste aversion (CTA)^5–9^. However, synaptic mechanisms underlying taste learning remains largely unknown.

Long-term potentiation (LTP) and long-term depression (LTD) of synaptic transmission are considered to be key synaptic mechanisms involved in learning and memory^10^. In the insular cortex, long-term plasticity at glutamatergic synapses has been well investigated^11–14^, and this form of synaptic plasticity is considered to be closely correlated with the taste learning^7,11,13,15,16^. In contrast, much less is known about long-term changes of synaptic efficacy at GABAergic synapses in the insular cortex. Because cortical interneurons dynamically control the excitability of pyramidal neurons^17^, plasticity of inhibitory synaptic transmission may exert major influences on insular excitability and function.

Long-term changes in synaptic efficacy can be caused by various synaptic mechanisms including presynaptic release properties and changes in subunit composition of postsynaptic receptors. As one of the potential mechanisms, retrograde messengers that are released from postsynaptic neurons play important roles in synaptic plasticity. The endocannabinoids (eCBs) and nitric oxide (NO) are well-known retrograde messengers. Thus far, the roles of eCBs and NO in plasticity at GABAergic synapses have been widely studied in the central nervous system (CNS)^18^. eCBs are synthesized postsynaptically in neurons following activation of G_q_-coupled receptors. In the CNS, eCBs decrease GABA release by binding to presynaptic cannabinoid 1 receptors (CB1Rs)^18,19^. In addition, eCBs have been demonstrated to contribute to induction of LTD at GABAergic synapses (LTD_GABA_) in the basolateral amygdala and also to be involved in amygdala-dependent extinction of aversive memories^20^. Furthermore, in the dorsomedial nucleus of the hypothalamus (DMH), GABAergic synapses exhibit eCB-dependent LTD in response to a prolonged high-frequency stimulation (HFS)^21^. NO is also produced postsynaptically in neurons in response to intracellular Ca^2+^ rises via activation of NMDA receptors. Unlike the action of eCBs, NO has been shown to increase GABAergic synaptic transmission in the CNS^22–24^. NO can play a crucial role in long-term potentiation at GABAergic synapses (LTP_GABA_) in the cerebellar Purkinje cells^25^, neonatal hippocampus^26^, deep cerebellar nuclei^27^, ventral tegmental area^28^ and DMH^21^. Thus, eCBs and NO exert contrasting actions in plasticity at GABAergic synapses. Interestingly, it has been shown that eCB and NO signaling interact to mediate plasticity at GABAergic synapses in the DMH^21^. However, until now, little is known about the roles of eCBs and NO in plasticity at GABAergic synapses in the insular cortex.

In the present study, we examined the plasticity at GABAergic synapses in layer V pyramidal neurons of the mouse insular cortex. We found that the plasticity at GABAergic synapses can be triggered by combined effects of eCB and NO signaling. The prolonged HFS causes LTD at GABAergic synapses (LTD_GABA_) which is mediated by eCB signaling. In contrast, the blockade of CB1Rs unmasks LTP_GABA_ which is mediated by NO production. In the presence of leptin which blocks eCB signaling, the prolonged HFS causes LTP_GABA_.

## Results

### A prolonged HFS induces LTD at GABAergic synapses in the insular cortex

**Figure 1:**
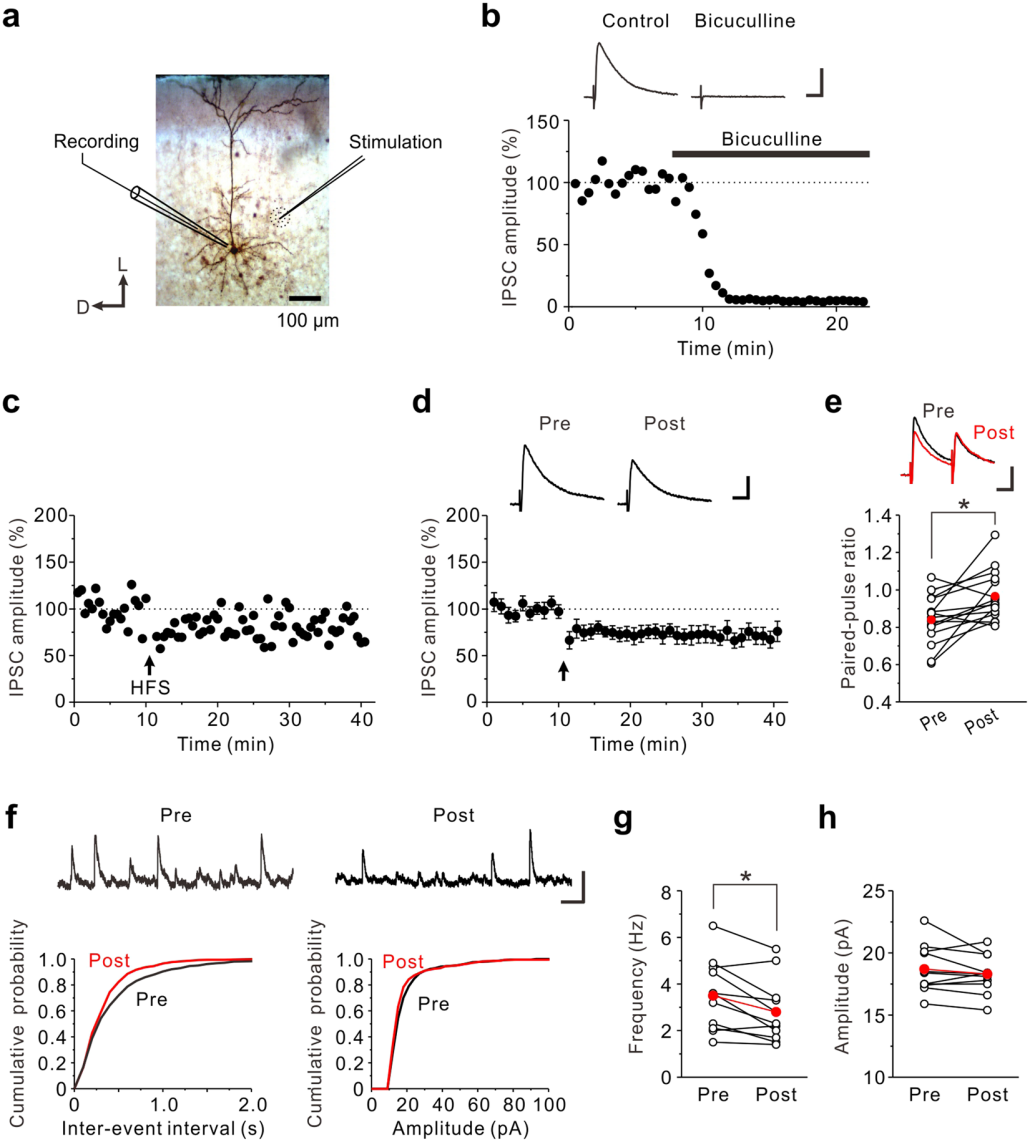
The prolonged HFS induces LTD_GABA_ in the insular cortex. (**a**) Biocytin-filled layer V pyramidal neuron; schematic illustration of the position of the recording and stimulation electrodes. D, Dorsal; L, lateral. (**b**) eIPSCs were almost completely abolised by 10 μM bicuculline. The inset shows averages of ten consecutive current traces before (Control) and 10–15 min after application of bicuculline. Scale bar represents 50 pA and 50 ms. (**c**) An example of LTD_GABA_ induced by the prolonged HFS. The prolonged HFS is indicated by an arrow. (**d**) Data from 13 neurons are summarized. The insets show averages of ten consecutive current traces before (pre) and 25–30 min after the prolonged HFS (post). The prolonged HFS is indicated by an arrow. Scale bar represents 50 pA and 50 ms. (**e**) Summary scatter plots of PPF (n = 15). *p < 0.05. The insets show averages of ten consecutive current traces before (pre) and 25–30 min after the prolonged HFS (post). Scale bar represents 50 pA and 50 ms. (**f**) Top: Representative traces of sIPSCs obtained before and after the prolonged HFS. Left: Cumulative inter-event interval distribution of sEPSCs obtained before and after the prolonged HFS. Right: Cumulative amplitude distribution of sEPSCs obtained before and after the prolonged HFS. Scale bar represents 50 pA and 200 ms. (**g**) Summary scatter plots of frequency of sIPSCs obtained before and after the prolonged HFS (n = 11). *p < 0.05. (**h**) Summary scatter plots of amplitude of sIPSCs obtained before and after the prolonged HFS (n = 11).

**Figure 2:**
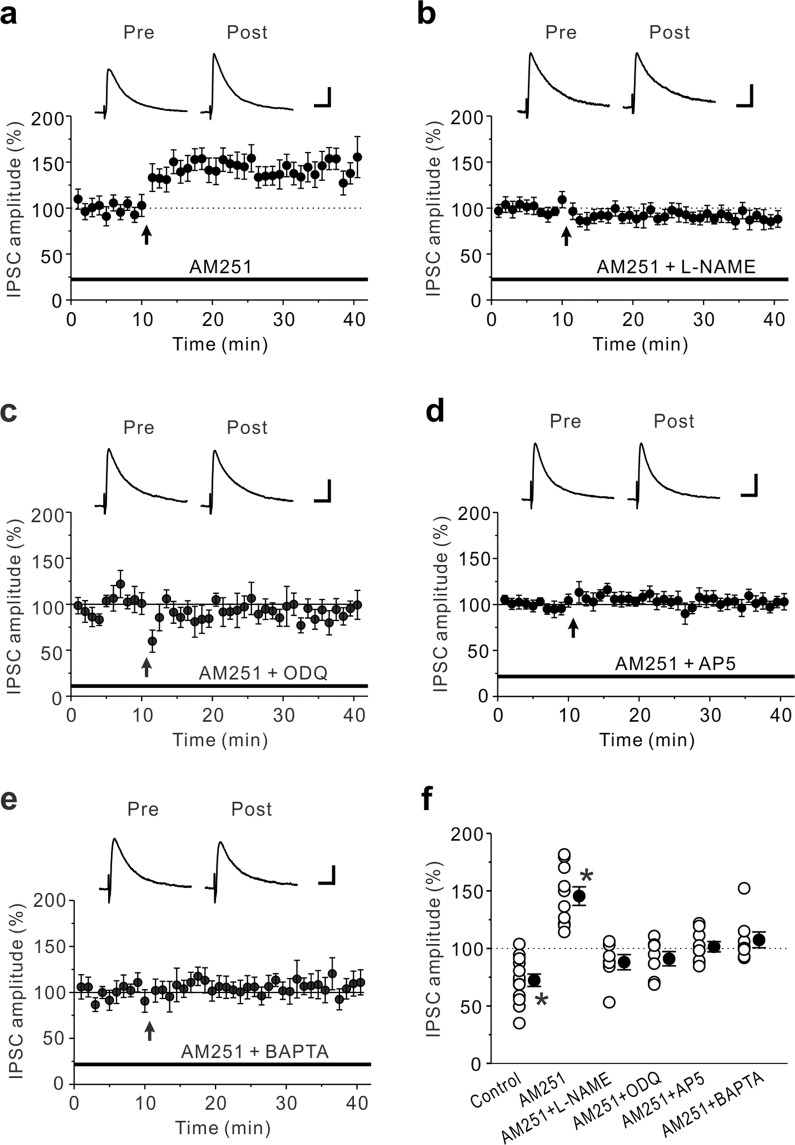
LTD_GABA_ induced by the prolonged HFS is shifted to LTP_GABA_ by CB1R blockade. (**a**) In the presence of 10 μM AM251, LTP_GABA_ was induced by the prolonged HFS (n = 10). (**b**-**e**) LTP_GABA_ caused by the prolonged HFS in the presence of 10 μM AM251 was almost completely abolished by 100 μM L-NAME (n = 7) (**b**), 10 μM ODQ (n = 7) (**c**), 50 μM AP-5 (n = 9) (**d**) and 10 mM BAPTA in the pipette solution (n = 8) (**e**). (**f**) Summary scatter plots of eIPSCs obtained from the experiments shown in Figs. 1d and 2a-e. Paired t-test, *p < 0.001 compared to the control response. (**a**)-(**e**) The insets show averages of ten consecutive current traces before (pre) and 25–30 min after the prolonged HFS (post). The prolonged HFS is indicated by an arrow. Scale bar represents 50 pA and 50 ms.

Whole-cell patch-clamp recordings were made from layer V pyramidal neurons of the mouse insular cortex (Fig. 1a). Evoked postsynaptic currents were recorded at a holding potential of 0 mV in the presence of 10 μM DNQX. We then examined whether evoked postsynaptic currents are solely mediated by GABAergic inputs onto layer V pyramidal neurons. Bath application of 10 μM bicuculline almost completely abolished the evoked postsynaptic currents (Fig. 1b, n = 5), suggesting that glutamatergic components were excluded under this recording condition. Next, we investigated how synaptic responses are modulated in response to a prolonged high-frequency stimulation (HFS, 100 Hz, 4 s, repeated four times with 15 s interval at a holding potential of 0 mV). The prolonged HFS induced LTD of GABAergic synapses (LTD_GABA_) in layer V pyramidal neurons of the insular cortex (72 ± 5% of baseline responses, n = 13; paired t-test: t(12) = −5.03, *p* < 0.001) (Fig. 1,d and 2f).

To determine the locus of LTD_GABA_, we examined whether and how the prolonged HFS modulates the paired-pulse ratio (PPR) and the frequency and amplitude of spontaneous IPSCs (sIPSCs). The paired-pulse stimulation is typically used to examine alterations in the probability of transmitter release^29^. The LTD_GABA_ caused by the prolonged HFS was associated with an increase in the PPF (pre: 0.84 ± 0.03; post: 0.97 ± 0.04, n = 15; paired t-test: t(14) = −3.16, *p* = 0.007) (Fig. 1e). After induction of LTD_GABA_ by the prolonged HFS, the frequency of sIPSCs was significantly decreased (pre: 3.5 ± 0.5 Hz; post: 2.8 ± 0.4 Hz, n = 11; paired t-test: t(10) = 3.20, *p* = 0.009) while the amplitude of sIPSCs was not altered (pre: 18.7 ± 0.6 pA; post: 18.3 ± 0.5 pA, n = 11; paired t-test: t(10) = 1.25, *p* = 0.240) (Fig. 1f,h). These results suggest that LTD_GABA_ caused by the prolonged HFS is expressed presynaptically.

### LTD_GABA_ is mediated by CB1Rs but its blockade shifts to LTP_GABA_

It has been shown that LTD_GABA_ caused by HFS for 4 s was mediated by endocannabinoids (eCBs) in the DMH^21^. We therefore examined whether eCBs are required for LTD_GABA_ in the insular cortex. In the presence of 10 μM AM251, a cannabinoid 1 receptor (CB1R) antagonist, the prolonged HFS induced LTP of GABAergic synapses (LTP_GABA_) (146 ± 8% of baseline responses, n = 10; paired t-test: t(9) = −4.91, *p* < 0.001) (Fig. 2a,f). This finding suggests that eCBs produced by the prolonged HFS induce LTD_GABA_ through acting on presynaptic CB1Rs while a retrograde messenger except eCBs contributes to LTP_GABA_.

Nitric oxide (NO) is known as a potential retrograde messenger in the insular cortex, in which neuronal NO synthase (nNOS)-immunoreactive neurons are distributed^30^. We then examined whether NO is necessary for the induction of LTP_GABA_. In the presence of 10 μM AM251 and 100 μM L-NAME (N^G^-nitro-L-arginine methyl ester, an inhibitor of nNOS synthase), LTP_GABA_ caused by the prolonged HFS was almost completely abolished (88 ± 7% of baseline responses, n = 7; paired t-test: t(6) = 1.81, *p* = 0.120) (Fig. 2b,f). The retrogradely released NO increases GABA release through activating a presynaptic cGMP signaling cascade^31^. We then examined whether soluble guanylyl cyclase (sGC) is necessary for the induction of LTP_GABA_. As expected, LTP_GABA_ caused by the prolonged HFS was completely abolished in the presence of 10 μM AM251 and 10 μM ODQ (1H-[1,2,4]oxadiazolo[4,3-a]quinoxalin-1-one, an inhibitor of sGC) (91 ± 6% of baseline responses, n = 7; paired t-test: t(6) = 1.89, *p* = 0.107) (Fig. 2c,f). These data suggest that activation of NO-sGC pathway leads to LTP_GABA_ in the insular cortex. Then, it is proposed that NO production in the insular cortex is caused by an increase in postsynaptic Ca^2+^ through activation of NMDA receptors^21,28^. We then examined whether and how LTP_GABA_ is modulated by the NMDA receptor antagonist AP-5, or the Ca^2+^ chelator BAPTA. In the presence of 10 μM AM251 and 50 μM AP-5, LTP_GABA_ caused by the prolonged HFS was completely abolished (102 ± 5% of baseline responses, n = 9; paired t-test: t(8) = −0.66, *p* = 0.526) (Fig. 2d,f). Loading the postsynaptic cell with 10 mM BAPTA also completely abolished the induction of LTP_GABA_ in the presence of 10 μM AM251 (107 ± 7% of baseline responses, n = 8; paired t-test: t(7) = −0.77, *p* = 0.465) (Fig. 2e,f). Taken together, these findings suggest that LTP_GABA_ is required for NO production which is generated by NMDA receptor-mediated postsynaptic Ca^2+^ rises and also suggest that NO produced following the prolonged HFS may act locally, mainly on NO-generating neurons^28^.

### Effects of a CB1R agonist and an NO donor on GABAergic synaptic transmission in the insular cortex

**Figure 3:**
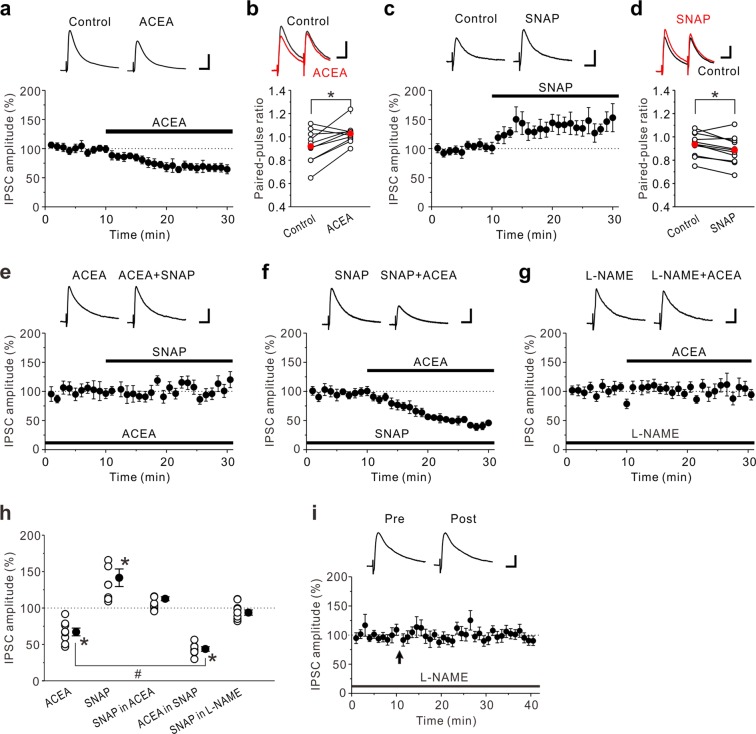
Effects of CB1R agonist and NO donor on eIPSCs in the insular cortex. (**a**) 3 μM ACEA decreased eIPSC amplitudes (n = 8). (**b**) Summary scatter plots of PPF before (Control) and during application of 3 μM ACEA (n = 10). ACEA increased PPF. (**c**) 200 μM SNAP increased eIPSC amplitudes (n = 8). (**d**) Summary scatter plots of PPF before (Control) and during application of 200 μM SNAP (n = 10). SNAP decreased PPF. (**e**) In the presence of 3 μM ACEA, 200 μM SNAP had no effect on eIPSC amplitudes (n = 8). (**f**) In the presence of 200 μM SNAP, 3 μM ACEA markedly decreased eIPSC amplitudes (n = 7). (**g**) In the presence of 100 μM L-NAME, 3 μM ACEA had no effect on amplitude of eIPSCs (n = 9). (**h**) Summary scatter plots of eIPSCs obtained from the experiments shown in (**a,c,e–g**). Paired t-test, *p < 0.02 compared to control response. Unpaired t-test, ^#^*p* < 0.01. (**i**) In the presence of 100 μM L-NAME, LTP_GABA_ caused by the prolonged HFS was completely abolished (n = 8). The insets show averages of ten consecutive current traces before (pre) and 25–30 min after the prolonged HFS (post). The prolonged HFS is indicated by an arrow. (**a,c,e–g**) The insets show averages of ten consecutive current traces before and 15–20 min after drug applications. (**a-g**,**i**) Scale bar represents 50 pA and 50 ms.

To explore the mechanisms that induce LTD_GABA_ and LTP_GABA_, we next investigated how a CB1R agonist ACEA and an NO donor SNAP modulate GABAergic synaptic transmission in the insular cortex. Bath application of 3 μM ACEA induced a significant reduction in the amplitude of evoked IPSCs (eIPSCs) (67 ± 8% of control responses, n = 8; paired t-test: t(7) = 6.14, *p* < 0.001) (Fig. 3a,h). The reduction of the eIPSC amplitude by ACEA was accompanied by an increase in the PPR (Control: 0.92 ± 0.04; ACEA: 1.03 ± 0.03, n = 10; paired t-test: t(9) = −2.98, *p* = 0.015) (Fig. 3b). In contrast, bath application of 200 μM SNAP induced a significant increase in the eIPSC amplitude (142 ± 18% of control responses, n = 8; paired t-test: t(7) = −3.53, *p* = 0.010) (Fig. 3c,h). The increase of the eIPSC amplitude by SNAP was accompanied by an decrease in the PPR (Control: 0.94 ± 0.03; SNAP: 0.89 ± 0.04, n = 10; paired t-test: t(9) = 2.85, *p* = 0.019) (Fig. 3d). These data support the evidence that activation of CB1Rs induces LTD_GABA_ while production of NO induces LTP_GABA_. Then, the question arises as to how eCB and NO signaling interact at GABAergic synapses in the insular cortex. In the DMH synapses, activation of CB1Rs at the presynaptic terminals suppresses the action of NO that increases GABA release^21^. In accordance with the previous findings, application of 200 μM SNAP in the presence of 3 μM ACEA, had almost no effect on the eIPSC amplitude (113 ± 15% of ACEA responses, n = 8; paired t-test: t(7) = −0.77, *p* = 0.467) (Fig. 3e,h). In contrast, application of 3 μM ACEA in the presence of 200 μM SNAP caused a large reduction in the eIPSC amplitude (44 ± 5% of control responses, n = 7; paired t-test: t(6) = 12.03, *p* < 0.001) (Fig. 3f,h). The reduction of the eIPSC amplitude caused by ACEA in the presence of SNAP was significantly larger than that caused by ACEA alone (unpaired t-test: t(13) = 3.75, *p* = 0.002) (Fig. 3h), suggesting that NO signaling enhances the effects of CB1R-mediated suppression of eIPSCs.

We next investigated whether NO production itself is required for the CB1R-mediated suppression of eIPSCs. In the presence of 100 μM L-NAME, 3 μM ACEA had no significant effect on the amplitude of eIPSCs (94 ± 4% of control responses, n = 9; paired t-test: t(8) = 0.97, *p* = 0.361) (Fig. 3g,h), indicating that NO production is necessary for the CB1R-mediated suppression of GABAergic synaptic transmission. As expected, LTD_GABA_ caused by the prolonged HFS was almost completely abolished in the presence of 100 μM L-NAME (97 ± 2% of baseline responses, n = 8; paired t-test: t(7) = 0.42, *p* = 0.689 (Fig. 3i). Taken together, NO production is critical for the induction of CB1R-mediated LTD_GABA_.

### Leptin-mediated suppression of CB1Rs unmasks LTP_GABA_

**Figure 4:**
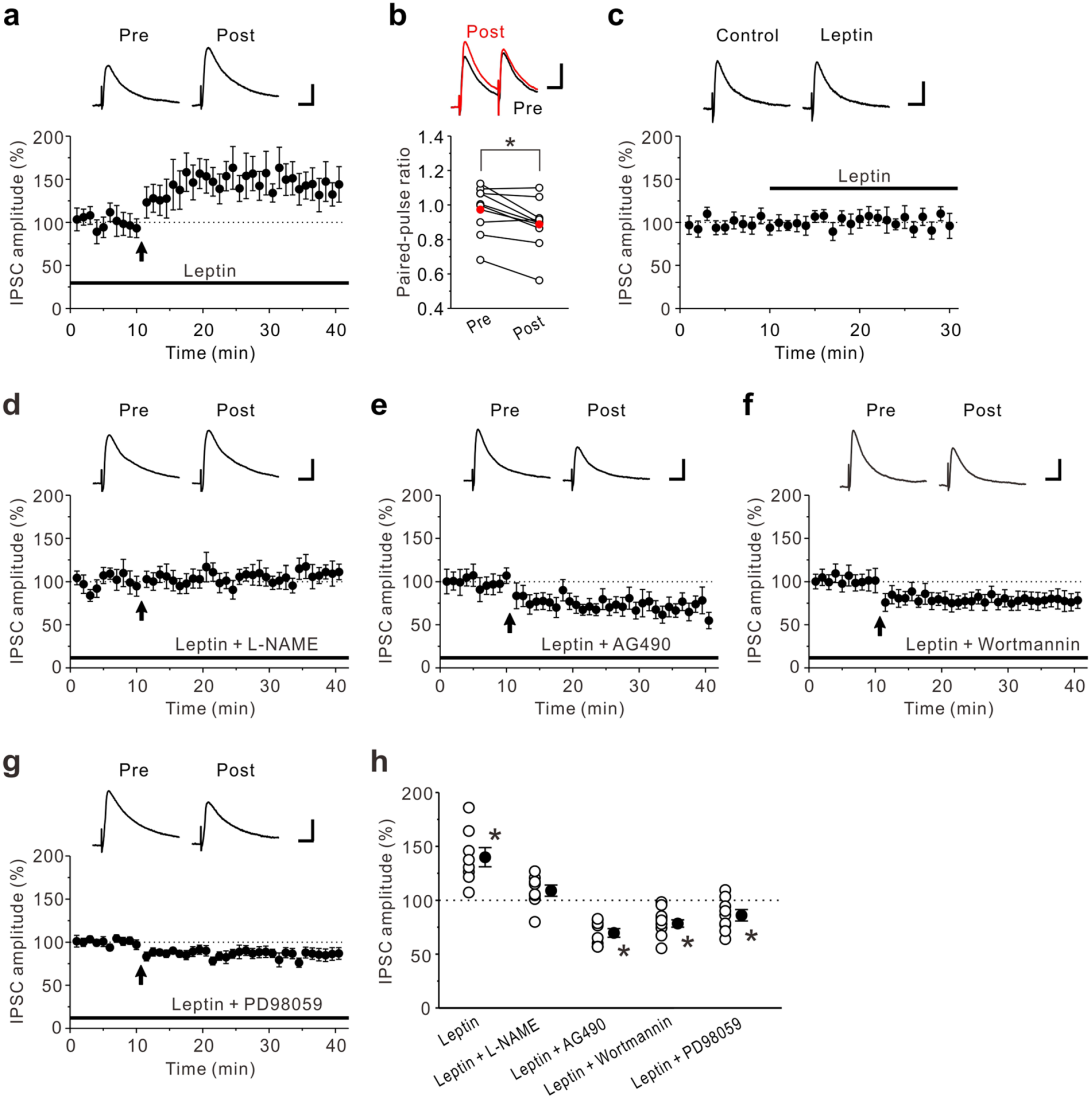
Leptin unmasks LTP_GABA_. (**a**) In the presence of 10 nM leptin, LTP_GABA_ was induced by the prolonged HFS (n = 8). (**b**) Summary scatter plots of PPF before and after the prolonged HFS in the presence of 10 nM leptin (n = 9). Leptin decreased PPF. (**c**) Leptin (10 nM) had no effect on eIPSCs (n = 9). The insets show averages of ten consecutive current traces before (Control) and 15–20 min after leptin application. (**d**) In the presence of 10 nM leptin and 100 μM L-NAME, the prolonged HFS had no effect on eIPSC amplitudes (n = 8). (**e**) In the presence of 10 nM leptin and 10 μM AG490, the prolonged HFS induced LTD_GABA_ (n = 8). (**f**) In the presence of 10 nM leptin and 200 nM wortmannin, the prolonged HFS induced LTD_GABA_ (n = 12). (**g**) In the presence of 10 nM leptin and 10 μM PD98059, the prolonged HFS induced LTD_GABA_ (n = 10). (**h**) Summary scatter plots of eIPSCs obtained from the experiments shown in (**a**,**d**–**g**). Paired t-test, *p < 0.04 compared to control response. (**a,c–g**) The insets show averages of ten consecutive current traces before (pre) and 25–30 min after HFS (post). (**a,d–g**) The prolonged HFS is indicated by an arrow. (**a–g**) Scale bar represents 50 pA and 50 ms.

There is evidence that leptin decreases food intake by reducing eCB levels^32^. We then examined the effects of leptin on LTD_GABA_ caused by the prolonged HFS in the insular cortex. Interestingly, the prolonged HFS induced LTP_GABA_ in the presence of 10 nM leptin in the insular cortex (140 ± 9% of control responses, n = 8; paired t-test: t(7) = −3.34, *p* = 0.012) (Fig. 4a,h). This was accompanied by an decrease in the PPF (pre-HFS: 0.97 ± 0.05; post-HFS: 0.89 ± 0.05, n = 9; paired t-test: t(8) = 3.32, *p* = 0.011) (Fig. 4b). These results suggest that leptin shifted LTD_GABA_ to LTP_GABA_ by inhibiting eCB signaling and that LTP_GABA_ was caused by a presynaptic mechanism. The effects of leptin on basal GABAergic synaptic transmission were also examined and we found that application of 10 nM leptin had almost no significant effect on the amplitude of eIPSCs (103 ± 3% of control responses, n = 9; paired t-test: t(8) = −0.30, *p* = 0.770) (Fig. 4c). These results indicate that leptin, at a concentration that does not affect basal GABAergic synaptic transmission, unmasks LTP_GABA_ in the insular cortex. To examine that LTP_GABA_ induced in the presence of leptin is dependent on NO signaling, we next investigated how LTP_GABA_ caused by the prolonged HFS is modulated in the presence of 10 nM leptin and 100 μM L-NAME. We found that LTP_GABA_ caused by the prolonged HFS was almost completely abolished in the presence of 10 nM leptin and 100 μM L-NAME (109 ± 5% of baseline responses, n = 8; paired t-test: t(7) = −1.16, *p* = 0.284 (Fig. 4d,h). These results suggest that LTP_GABA_ induced in the presence of leptin is dependent on NO-mediated facilitation of GABAergic synaptic transmission.

We further examined the signal transduction mechanisms by which leptin unmasks LTP_GABA_. Leptin binds leptin receptors which interact with JAK2 (janus kinase 2) via intracellular docking sites^33^. Activation of JAK2 can lead to the activation of downstream kinase cascades, including mitogen-activated protein kinase (MAPK) and phosphatidylinositol 3 kinase (PI3K)^33^. We first tested the effects of a tyrosine kinase inhibitor AG490, which blocks JAK2 phosphorylation on LTP_GABA_ induced in the presence of leptin. In the presence of 10 nM leptin and 10 μM AG490, the prolonged HFS induced LTD_GABA_ (70 ± 4% of baseline responses, n = 8; paired t-test: t(7) = 5.28, *p* = 0.001) (Fig. 4e,h). We next examined the effects of a PI3K inhibitor wortmannin, on LTP_GABA_ induced in the presence of leptin. In the presence of 10 nM leptin and 200 nM wortmannin, the prolonged HFS induced LTD_GABA_ (78 ± 3% of baseline responses, n = 12; paired t-test: t(11) = 5.04, *p* < 0.001) (Fig. 4f,h). Furthermore, we examined the effects of a MAPK inhibitor PD98059, on LTP_GABA_ induced in the presence of leptin. In the presence of 10 nM leptin and 10 μM PD98059, the prolonged HFS induced LTD_GABA_ (86 ± 5% of baseline responses, n = 10; paired t-test: t(9) = 2.47, *p* = 0.036) (Fig. 4g,h). These results suggest that JAK2-PI3K and JAK2-MAPK signaling pathways are involved in LTP_GABA_ induced in the presence of leptin.

## Discussion

We have shown that the prolonged HFS induced LTD_GABA_ in the insular cortex. This synaptic plasticity is triggered by NO production via activation of postsynaptic NMDA receptors. In addition, this synaptic plasticity is required for eCBs as the retrograde messenger and is maintained by persistent presynaptic alteration of GABA release nearby synaptic terminals. In the presence of the CB1R antagonist AM251, the same stimuli induced LTP_GABA_, which was mediated by NO production via activation of NMDA receptors. The NO signaling was indispensable for induction of LTD_GABA_ in the insular cortex. Furthermore, in the presence of leptin, a hormone that regulates food intake, energy homeostasis, learning and memory, the prolonged HFS caused LTP_GABA_. Our findings will impact on the study of the synaptic mechanisms for taste learning in the gustatory insular cortex.

We provide the evidence that the interaction between eCB and NO signaling plays a key role in modulating plasticity at GABAergic synapses in the insular cortex. We found that activation of eCB signaling by the prolonged HFS suppresses NO-mediated LTP_GABA_. This is demonstrated by the observations that when the prolonged HFS is applied to insular cortical slices in the presence of the CB1R antagonist AM251, NO-mediated LTP_GABA_ was unmasked in the insular cortex (Fig. 2a). In the DMH, HFS for 1 s induces NO-mediated LTP_GABA_ while HFS for 4 s induces eCB-mediated LTD_GABA_^21^. These results may suggest that the shorter duration of HFS causes the production of NO over eCBs, whereas the longer duration of HFS produces both NO and eCBs and activation of eCB signaling suppresses NO-mediated LTP_GABA_. Although the production of both NO and eCBs are triggered by postsynaptic Ca^2+^ rises^19^, it is possible that NO may readily permeate the presynaptic membrane compared to eCBs and acts faster than eCBs as the retrograde signal. This is because the membranes do not have a barrier to gases such as NO while the spread of eCBs is confined to extracellular space between cells^34^.

Our data showed that NO signaling is essential for eCB-mediated LTD_GABA_ in the insular cortex. When the production of NO was blocked by L-NAME, the prolonged HFS did not induce LTD_GABA_ (Fig. 3i) and the CB1R agonist ACEA did not decrease the eIPSC amplitude (Fig. 3g). By contrast, when the production of NO was enhanced by the NO donor SNAP, the ACEA-induced decrease in eIPSC amplitude was significantly enhanced (Fig. 3f). These observations are agreement with previous reports that the inhibition of NO signaling blocked eCB-mediated synaptic plasticity in other brain areas^35–37^. Although it is still unclear how eCB and NO signaling interact with each other, several possible mechanisms are postulated. The finding that the NOS inhibitor blocked the induction of eCB-mediated LTD at parallel fiber synapses suggests that NO acts downstream of CB1R activation in the cerebellum^37^. In contrast, the effects of the NO signaling pathway on depolarization-induced suppression of inhibition in CA1 pyramidal cells were blocked by the CB1R antagonist AM251, suggesting that NO acts upstream of CB1R activation in the hippocampus^36^. However, we cannot rule out the possibility that NO enhances production of eCBs at postsynaptic sites.

We also found that activation of CB1Rs suppresses the NO-induced enhancement of GABAergic synaptic transmission. This is demonstrated by the observations that the NO donor SNAP had no effect on the eIPSC amplitude in the presence of the CB1 agonist ACEA (Fig. 3e). Similarly, it has been reported that SNAP failed to enhance GABAergic synaptic transmission in the DMH in the presence of the CB1 agonist WIN 55,212-2^21^. Although it was demonstrated that activation of CB1Rs inhibits NOS activity in the rat cerebellar granule cells^38^, the mechanisms of the CB1-mediated suppression of the NO action are not well understood. Our data and the previous results indicate that activation of CB1Rs may repress NO signaling at the level of guanylyl cyclase.

The gustatory insular cortex processes not only multiple taste attributes such as texture, viscosity and modality^39^ but also taste hedonics and memories^40,41^. The GABAergic activity of the gustatory insular cortex has been implicated in controlling the ability of organisms to encode information regarding sensory stimuli during memory acquisition, consolidation and retrieval. The administration of the GABA_A_ receptor-selective agonist muscimol into the rat gustatory insular cortex impaired both acquisition and retrieval of taste memory^42^. Furthermore, the microinjection of muscimol into the rat agranular insular cortex suppressed palatability-driven feeding without affecting drinking^43^. These observations indicate that the GABAergic inhibitory system apparently contributes to modifying taste memory. In food deprived animals in which corticosteroids-mediated loss of CB1 signaling occurs, GABAergic synapses in the DMH exhibit LTP_GABA_ in response to HFS^21^. In addition, stressed animals are known to exhibit an enhancement of CTA learning^44^. Thus, when CB1 signaling is impaired by stress or other factors, it is possible that NO-mediated LTP_GABA_ is readily induced by HFS in the gustatory insular cortex. Although the functions of NO in taste learning are not fully understood, it was demonstrated that injection of the NO donor sodium nitroprusside (SNP) produced CTA in rats^45^.

Several evidence suggests that eCB-mediated GABAergic synaptic plasticity occurs *in vivo*, and inhibiting eCB signaling exhibits deleterious effects on learning and memory^46,47^. For example, mice lacking CB1Rs show an impairment of eCB-mediated LTD_GABA_ in the basolateral amygdala together with a deterioration of short-term and long-term extinction in auditory fear memory^20^. The CB1Rs in the gustatory insular cortex have been demonstrated to play pivotal roles in CTA learning and memory. Activation of CB1Rs in the gustatory insular cortex impairs acquisition and reconsolidation of CTA memory but not its extinction, whereas interfering with CB1Rs enhances acquisition and inhibits extinction without having no apparent effect on reconsolidation^48^. These observations indicate that LTD_GABA_ induced by the prolonged HFS could contribute to extinction of taste learning.

Leptin is a peptide hormone which is associated with regulation of food intake and energy metabolism via activation of hypothalamic neurons^49^. However, the effects of leptin are not confined to the hypothalamus because increasing evidence indicates that leptin receptors are widely expressed in the brain and that leptin mediates central actions such as learning and memory^50^. It has been shown that deficiency of leptin alters brain functions such as memory processes, which can be recovered by leptin administration^50^. Leptin receptors are also expressed in the gustatory insular cortex^51,52^, suggesting that these receptors are involved in taste learning. In the present study, we found that leptin shifted LTD_GABA_ to LTP_GABA_, presumably by inhibiting eCB signaling (Fig. 4a). Considering that blockade of CB1Rs facilitates acquisition of CTA memory and suppresses its extinction^48^, it is possible that leptin increases acquisition of CTA memory while decreasing its extinction. Furthermore, we found that the inhibitory effects of leptin on eCB-mediated LTD_GABA_ were abolished by inhibitors of JAK2, PI3K and MAPK signaling pathways (Fig. 4e–g). It is reported that leptin suppresses release of eCBs by inhibiting voltage-gated Ca^2+^ currents (VGCCs) via JAK2-MAPK pathway in mouse perifornical lateral hypothalamus neurons^53^. Furthermore, it is shown that leptin inhibits VGCCs in neuropeptide Y neurons via JAK2-MAPK pathway, whereas it increases VGCCs in proopiomelanocortin neurons via JAK2-PI3K pathway^54^. Although our study did not examine the effects of leptin on VGCCs, it is likely that leptin suppresses eCB signaling by inhibiting VGCCs via both JAK2-MAPK and JAK2-PI3K pathways in the insular cortex.

In the present study, we for the first time demonstrate the plasticity of GABAergic synapses in the insular cortex, which can be triggered by combined effects of eCBs and NO. However, it should be considered that the rules that apply to the synaptic plasticity *in vivo* are not necessarily the same as those found in *in vitro* studies. This is because it is well known that drug treatment *in vivo* can cause some behavioral changes in animals. In addition, there is a possibility that recordings from brain slices *in vitro* lose the effects of drugs for the slice incubation for long time in the aCSF. Therefore, it would be necessary to examine the mechanisms of the plasticity of GABAergic synapses *in vivo*. Future intensive studies into more detailed mechanisms of plasticity at GABAergic synapses and their behavioral relevance would be helpful for understanding the synaptic basis for the involvement of the insular cortex in higher brain functions including taste memory.

## Methods

All experiments were carried out in accordance with the European Communities Council Directive of 2010/63/EU. All experimental protocols were approved by the animal ethics committees of the Osaka University Graduate School of Dentistry for the care and use of laboratory animals, and all experiments were performed in accordance with the relevant guidelines. All efforts were made to minimize the suffering as well as the number of animals.

### Slice preparation

Male C57BL/6J mice at 3–5 weeks old were used in the experiments and they were purchased from Japan SLC (Hamamatsu, Japan). They were anesthetized with isoflurane, and the brain was quickly removed from the skull and immersed in ice-cold modified artificial cerebrospinal fluid (aCSF) composed of 210 mM sucrose, 2.5 mM KCl, 2.5 mM MgSO_4_, 1.25 mM NaH_2_PO_4_, 26 mM NaHCO_3_, 0.5 mM CaCl_2_ and 50 mM d-glucose. With a microslicer (Linearslicer Pro 7, Dosaka EM, Kyoto, Japan), coronal sections of 300 μm thickness including the gustatory insular cortex were cut^55^. Slices were incubated at 32 °C for 30 min in 50% modified aCSF and 50% normal aCSF (pH 7.3) composed of 126 mM NaCl, 3 mM KCl, 1 mM MgSO_4_, 1.25 mM NaH_2_PO_4_, 26 mM NaHCO_3_, 2 mM CaCl_2_ and 10 mM d-glucose. The slices were then placed in normal aCSF at room temperature (20–24 °C). Normal aCSF was continuously gassed with a mixture of 95% O_2_–5% CO_2_.

### Whole-cell patch-clamp recordings

Whole-cell recordings were performed similar to our previous study^56^. Brain slices including the insular cortex were transferred to the recording chamber and perfused with normal aCSF at a flow rate of 2 ml/min. Using MultiClamp 700B Amplifier (Molecular Devices, Foster City, CA), whole-cell recordings were made from visually identified pyramidal neurons in layer V of the gustatory insular cortex^55^. Neurons were visualized using differential interference contrast microscopy (BX-51WI; Olympus, Tokyo). All electrophysiological experiments were performed at 30–32 °C.

When inhibitory postsynaptic currents (IPSCs) were recorded, the recording pipettes (3–5 MΩ) were filled with solution containing 130 mM Cs-gluconate, 10 mM CsCl, 2 mM MgCl_2_, 2 mM ATP-Na_2_, 0.4 mM GTP-Na_3_, 10 mM HEPES and 0.2 mM EGTA; pH 7.3, adjusted with CsOH^57^. Biocytin (2 mg/ml) was added in the pipette solution for later visualization and morphological identification of neurons. In some experiments, 10 mM 1,2-Bis(2-aminophenoxy)ethane-N,N,N’,N’-tetraacetic acid (BAPTA) was included in the recording pipettes. The Cl^−^ equilibrium potential (E_Cl_) was calculated to be −57 mV. IPSCs were recorded at a holding potential of 0 mV. eIPSCs were recorded from layer V pyramidal neurons, and stimuli were delivered by a monopolar tungsten stimulating electrode placed within layer V in the agranular insular cortex (~200 µm along the apical dendrite of the cell). IPSCs were evoked by repetitive stimuli (duration is 100 μs, intensity is adjusted to induce EPSCs with an amplitude of 100–200 pA) at 0.033 Hz. eIPSCs were recorded in the presence of 10 μM DNQX (6,7-dinitroquinoxaline-2,3-dione, a non-NMDA receptor antagonist). Paired-pulse responses of eIPSCs were obtained by applying a pair of synaptic stimuli 50 ms apart. For high-frequency stimulation (HFS), stimuli were applied at 100 Hz for 4 s, repeated 4 times at 15 s interval. Access resistance was 15–20 MΩ and was monitored throughout the experiment. Data were discarded if access resistance changed more than 15% during an experiment.

Effects of HFS on spontaneous IPSCs (sIPSCs) were examined in the presence of 10 μM DNQX and 50 μM AP-5 (dl-2-amino-5-phosphonopentanoic acid, an NMDA receptor antagonist). The sealing resistance was usually more than 10 GΩ. The membrane potential values were corrected for the liquid junction potential (10 mV) between the internal solutions (negative) and the extracellular solution. Signals were low-pass filtered at 2 kHz (4-pole Bessel filter) and digitized at a sampling rate of 2–10 kHz (1440 A, Molecular Devices).

### Biocytin labeling

All steps were performed as reported previously^56^. After recordings, brain slices were fixed in 4% paraformaldehyde in 0.1 M phosphate buffer (PB, pH 7.4). Sections were rinsed in 0.1 M PB containing 0.3% Triton X-100 and then treated with 0.5% H_2_O_2_ for 1 h. After thoroughly washing with 0.1 M PB, the sections were incubated overnight at 4 °C in avidin-biotinylated enzyme complex (ABC reagent, 1:100; Vector Laboratories, Burlingame, CA) following the instructions of the manufacturer. For the detection of signals, the sections were preincubated in 0.05% 3-3′-diaminobenizidine (DAB; Sigma-Aldrich) in 0.1 M PB for 10 min and then transferred to the same solution containing 0.03% H_2_O_2_. The reaction was stopped by rinsing the slices in 0.1 M PB. Stained sections were dehydrated and coverslipped and then imaged using a light microscope (BX-40; Olympus).

### Drug application

The following drugs were used in the present study. The CB1 receptor agonist (ACEA, 3 μM); the inhibitor of the janus kinase 2 protein (AG490, 10 μM); the CB1 receptor antagonist (AM251, 10 μM); the NMDA receptor antagonist, dl-2-amino-5-phosphonopentanoic acid (AP-5, 50 μM); the GABA_A_ receptor antagonist, bicuculline (10 μM); the non-NMDA receptor antagonist, 6,7-dinitroquinoxaline-2,3-dione (DNQX, 10 μM); the nitric oxide synthase inhibitor, N^G^-nitro-L-arginine methyl ester (L-NAME, 100 μM); the peptide hormone, leptin (10 nM), the soluble guanylyl cyclase (sGC) inhibitor, 1H-[1,2,4]oxadiazolo[4,3-a]quinoxalin-1-one (ODQ, 10 μM); the mitogen-activated protein kinase (MAPK) inhibitor (PD98059, 10 μM); the nitric oxide (NO) donor, S-nitroso-N-acetylpenicillamine (SNAP, 200 μM); the phosphoinositide 3 kinase inhibitor (wortmannin, 200 nM). ACEA, AG490, AM251, AP-5, bicuculline, L-NAME, leptin, ODG, PD98059, SNAP and wortmannin were bath applied. Drugs were applied for at least 15 min before HFS or ACEA/SNAP applications or as otherwise stated in the text. BAPTA was included in the patch-pipette solution. ACEA, AM251, AP-5 were obtained from Tocris Bioscience (Bristol, UK). Leptin was obtained from Peptide Institute (Osaka, Japan). AG490, PD98059 and wortmannin were obtained from Abcam (Cambridge, UK). SNAP was obtained from Wako Pure Chemicals (Osaka, Japan). All other chemicals were obtained from Sigma-Aldrich (St Louis, MO).

### Statistical analysis

Data analysis was carried out similar to our previous study^56^. Synaptic strength was measured as the change in eIPSC amplitude when comparing an average eIPSC amplitude measured in a period of 25–30 min after HFS to a baseline eIPSC amplitude measured in the last 5 min of control recordings. Effects of ACEA, SNAP and leptin on eIPSC amplitude were evaluated as the change in eIPSC amplitude when comparing an average eIPSC amplitude measured in a period of 15–20 min after drug applications to a baseline eIPSC amplitude measured in the last 5 min of control recordings. Frequency and amplitude of sIPSCs were analyzed using Minianalysis software (Synaptosoft, Decatur, GA). To evaluate effects of HFS on sIPSCs, the percentage changes in frequency and amplitude of sIPSCs were used and were determined by comparing the frequency and amplitude of sIPSCs measured in a period of 25–30 min after HFS to the baseline frequency and amplitude of sIPSCs measured in the last 5 min of control recordings.

Numerical data were expressed as the mean ± S.E. The statistical significance was assessed using unpaired or paired Student’s *t*-test. Where described in text, Student’s t-tests were used to compare responses from several neurons. Student’s t-test was used when the data showed the normal distribution. Statistical results were given as a precise *p* value, except when *p* was very small (*p* < 0.001). *p* < 0.05 was considered statistically significant.
